# Temporal and geographic analyses of colorectal cancer screening during and after the COVID-19 pandemic in a federally qualified health center

**DOI:** 10.1371/journal.pone.0345248

**Published:** 2026-03-24

**Authors:** Gloria D. Coronado, John F. Dickerson, Ming-Hsiang Tsou, Namrata Shivaprakash, Ana G. Rosales, Jackson L. Voelkel, Meisha A. Whyte, Elizabeth Shuster, Amanda F. Petrik, Josheili Y. Llavona-Ortiz, Charisma L. Jenkins, Carolyn M. Rutter, Anne L. Escaron

**Affiliations:** 1 University of Arizona Cancer Center, Tucson, Arizona, United States of America; 2 Kaiser Permanente Center for Health Research, Portland, Oregon, United States of America; 3 San Diego State University, San Diego, California, United States of America; 4 AltaMed Health Services Corporation, Los Angeles, California, United States of America; 5 Kaiser Foundation Health Plan, Inc., Portland, Oregon, United States of America; 6 Fred Hutchinson Cancer Center, Seattle, Washington, United States of America; Auburn University, UNITED STATES OF AMERICA

## Abstract

**Background:**

The COVID-19 pandemic caused reductions in cancer screening services. We assessed the pandemic’s impact on colorectal cancer screening in a large diverse federally qualified health center (FQHC) in Los Angeles, CA.

**Methods:**

We used interrupted time series regression to estimate trends in monthly colorectal cancer screening rates for four relevant COVID-19 pandemic periods: pre-pandemic (March 2018 – February 2020); early-pandemic (March – December 2020); vaccine-era (January 2021– May 2023); and post-pandemic (June 2023 – May 2024). We plotted spatial distribution patterns of screening across census tracts.

**Results:**

Participants were 83,430 unique individuals (55% male; 80% Hispanic) ages 50–75. Average monthly colorectal cancer screening rates dropped from 9.3% pre-pandemic to 5.9% early-pandemic. Monthly screening rates in the vaccine era (7.5%) never returned to pre-pandemic levels and further declined in the post-pandemic era (6.7%; p trend = 0.09). Screening rates were consistently higher for males, ages 65–75, Hispanic individuals, and Spanish-preferring individuals in both pre-COVID (March 2018-Feb 2020) and post-COVID (July 2020-May 2024) periods. Increases in stool-based testing aligned with mailed outreach campaigns.

**Conclusions:**

Monthly post-pandemic screening rates never reached pre-pandemic levels and declined from 2023 to 2024. Sharp increases in stool-based testing coincided with mailed outreach events, highlighting the importance of home-based screening methods during disruptive events.

**Impact:**

Our findings can help shape healthcare response strategies to reduce screening delays in the context of future natural disasters.

## Introduction

The COVID-19 pandemic led to major disruptions in healthcare systems, affecting the delivery of preventive care services, including colorectal cancer screening. Care suspensions and limited capacity for colonoscopy delivery prompted national organizations to recommend increased use of stool-based testing options, followed by colonoscopy for those with abnormal test results [1,2]. Screening patterns following the pandemic, as documented in US national surveillance systems and among commercially insured adults, aligned with these recommendations. Star and colleagues found comparable past-year colorectal cancer screening prevalence when comparing 2021–2019 National Health Interview Survey (NHIS) data, observing that this stability resulted from a 44% increase in past-year home-based stool blood testing (from 7.0% to 10.3%; adjusted Prevalence Ratio [aPR], 1.44; 95% CI, 1.31 to 1.58) that counterbalanced a 12% decrease in colonoscopy use (from 15.5% to 13.8%; aPR, 0.88; 95% CI, 0.83 to 0.95) [3]. By 2023, NHIS data showed colorectal cancer screening rates surpassing pre-pandemic levels [4]. Similarly, an evaluation of pre-pandemic (January 1, 2017, to February 28, 2020) and post-pandemic (July 1, 2020, to December 31, 2024) colorectal cancer screening trends among Blue Cross and Blue Shield commercial enrollees showed decreased post-pandemic colonoscopy and fecal immunochemical testing rates but increased FIT-DNA testing rates [5].

Federally Qualified Health Centers (FQHCs) played a critical role during the COVID-19 pandemic by delivering essential testing, vaccinations, and primary care services to vulnerable populations [6]. Approximately 30 million individuals across the United States and its territories receive care at more than 14,000 FQHC delivery sites every year [7]. While FQHC colorectal cancer screening rates had consistently improved from 2014 to 2019, these rates remain substantially below the national average: 25 percentage points lower (43% vs. 68% in 2019) [8,9]. Similar to other health systems, FQHCs faced pandemic-related closures of clinic sites, reductions in staffing, decreases in clinic volumes for routine care, and the implementation of mitigation strategies such as telehealth and social distancing [10–12]. These challenges were compounded for FQHCs given their mission to serve predominantly low-income and low-literacy populations. Therefore, understanding COVID-related impacts in this setting is critically important [10,11].

Limited prior evaluations of colorectal cancer screening trends in FQHCs showed a pattern of decline immediately following pandemic-related lockdowns and incomplete recovery through 2022. McGrath and colleagues examined colorectal cancer screening among patients receiving care in nearly 200 FQHCs (465,778 age-eligible patients) from January 2019 to December 2022, reporting a 6.6 percentage-point rate decline from 2019 (45.2%) to 2020 (38.6%), followed by a 4.3 percentage-point recovery from 2020 to 2022 (42.9%) [13]. An analysis by Zhao and colleagues of 1,701 FQHCs revealed deepening disparities, with clinics primarily serving Latino or Black patients experiencing more severe disruptions and slower rates of recovery [9]. While these studies and two prior studies examined colorectal cancer screening rates in safety-net health systems, they are limited to data through December 2022, providing sparse information about long-term rebound in colorectal cancer screening rates [9,13–15]. Our study extends the evidence base through 2024, examines patterns across demographic subgroups, and uses geospatial methods to identify areas of geographic vulnerability. We present analyses of electronic health record (EHR) data from the nation’s largest FQHC which operates sites across Los Angeles County and Orange County; our findings can inform efforts to optimize recovery from future natural disasters.

## Materials and methods

### Study setting

The study was conducted as a partnership among Kaiser Permanente Center for Health Research, the University of Arizona, and a large FQHC in Southern California. The health center operates 27 medical clinics and served more than 290,000 patients in 2024, including about 71,000 patients ages 50–75. More than 97% of patients ages 50–75 had English (33%) or Spanish (65%) listed as their preferred language. The health center uses the InSure® ONE™ Fecal Immunochemical Test (FIT; Clinical Genomics; Bridgewater, NJ), which requires collection of two specimens from a single stool sample. FITs are processed at a reference laboratory (Quest) and results are transferred to the health center through a direct EHR interface. Patients with an abnormal stool test result and patients at high risk or reporting symptoms of colorectal cancer are referred for colonoscopy.

All procedures and intervention materials were reviewed and approved by the Kaiser Permanente Interregional Institutional Review Board (IRB # 1752845), with ceding agreements from the health center, and the University of Arizona. The study obtained a waiver of informed consent and authorization for use of protected health information, given the minimal risk posed to patients.

### Data measures

De-identified patient data was initially accessed on January 19, 2024, and was later updated on April 16, 2025. Using the EHR, we identified patients each month who were aged 50–75 and had a clinic visit (in-person or telemedicine visit where health topics were discussed) within the past two years. We excluded patients who ever had a personal history of colorectal cancer, colectomy, or metastatic tumor; history of dementia, Parkinson disease, or hospice care (including home oxygen use and shortness of breath at rest); or colorectal disease (i.e., Crohn’s disease, ulcerative colitis). Each month, patients were excluded if they were current with colorectal cancer screening recommendations (i.e., colonoscopy in the past 10 years, FIT in the past year, FIT-DNA in the past 3 years, or sigmoidoscopy in the past 5 years). The remaining patients constituted our eligible patients. Monthly screening rates were calculated by dividing the number of patients who received any colorectal cancer screening service (colonoscopy, FIT, or FIT-DNA) in a given month by the total number of eligible patients in the same month.

For the purposes of excluding patients who sought care at the health center only for a COVID test or vaccination, we limited our analyses to patients who were enrolled in a managed-care Medicaid program or were assigned a primary care provider. The FQHC routinely empanels Medicaid managed-care patients and uninsured patients to a primary care site and provider if they do not select one on their own. Similarly, commercially insured and Medicare patients are routinely assigned to a primary care provider within a couple of visits.

### Statistical analysis

The relevant analysis sample for the primary outcome (stool testing and colonoscopy proportions) included all people who were eligible for screening. The unit of analysis is calendar month. We assessed screening outcomes using interrupted time series regression to estimate changes in monthly screening rates following onset of the COVID-19 pandemic. We estimated the level and slope of screening rates over time prior to and following the start of the COVID-19 pandemic. The change in level provides an estimate of the immediate effect of the COVID-19 pandemic, and the change in slope provides an estimate of the effect across time. We stratified screening outcomes by screening modality (i.e., any colorectal cancer screening tests, stool-based tests, FIT, FIT-DNA, colonoscopy). We use line graphs to plot monthly screening rates of stool-based testing and colonoscopy, and to plot these rates further stratified by EHR-derived patient demographic variables (i.e., sex [male vs. female], age [50–64 vs. 65–75], ethnicity [Hispanic vs. non-Hispanic], and language preference [English vs. Spanish]). The global trend was calculated using linear regression analysis, with test type rates serving as dependent variables and time as the independent variable. These models incorporated adjustments for potential autocorrelation. In post-hoc analysis, given steep rises in stool-based testing following mailed FIT outreach campaigns, we assessed differences across COVID-19-related timepoints in stool-based testing with and without data from the months that the large, annual mailed FIT outreach program took place. This included July and August 2018, July and August 2019, June and July 2020, and July and August 2021, and August and September 2022. We did not apply month-specific exclusions for 2023 or 2024 because multiple mailing occurred in those years.

We include 75 time points (5 years; March 2018 – May 2024) in total: 24 pre-pandemic-months (March 2018 – February 2020), 10 early-pandemic-months (March – December 2020), 29 vaccine availability months (January 2021– May 2023), following vaccine approval, and 12 post-pandemic months (June 2023 – May 2024), after the COVID-19 national emergency declaration was lifted. This number of timepoints exceeds the range of 12 points prior and post intervention which are necessary to detect modest effects in segmented regression analyses used to inform policy at the system level [16–18]. Statistical analyses were completed using SAS 9.4.

### Geo-spatial analysis

We used geographic information system (GIS) tools to map average screening uptake by screening test modality in the geographic region served by our health center and across the COVID-19-relevant time intervals. Home addresses for all eligible patients were geocoded at the census tract level using ArcGIS Pro to convert addresses to latitude/longitude coordinates. Using R Project for Statistical Computing and US Census Bureau population data, these geographic points were aggregated to census tracts where colorectal cancer screening proportions were calculated. Data from census tracts with fewer than 6 patients were suppressed as “unavailable” to protect privacy. ArcGIS Pro was then used to visualize dynamic changes in colorectal cancer screening completion (both stool-based tests and colonoscopy) among eligible patients across four COVID-19 time intervals. Average monthly colorectal cancer screening proportions were weighted according to length of time in that time interval. S1 Fig illustrates the cohort population density (health center patients) across the four COVID-19 time intervals. We observed relatively high patient density (darker color regions) in areas matching the spatial patterns of high densities of Hispanic and low-income families in LA and Orange counties. High patient density areas are also very close to the locations of clinics (represented by red dots). These maps show no significant changes of patient cohort density and spatial distribution across the study time intervals.

## Results

We identified a total of 83,430 unique individuals (1,707,538 observations): 15,734 individuals (377,617 observations) in the pre-pandemic interval, 23,305 (233,046 observations) in the early-pandemic interval, 26,157 (758,539 observations) in the vaccine interval, and 11,667 (338,336 observations) in the post-pandemic interval (Table 1). Demographic characteristics were similar across pandemic-relevant phases. Overall, participants were largely 50–59 years old (58.5%), male (55%), and Hispanic (80.7%). Based on EHR data, Spanish was the preferred language for more than half (63.5%) of participants and nearly half (47.7%) had more than 3 clinic visits in the prior year.

**Table 1:** Demographic characteristics of colorectal cancer screening-eligible managed care and empaneled health center patients ages 50-75, by COVID-related time interval.

Characteristic		COVID-related time interval			
	Overall (N = 76,862)	Pre-pandemic (March 2018 – February 2020) 24 months (N = 15,734)	Early-pandemic (March 2020- December 2020) 10 months (N = 23,305)	Vaccine-era (January 2021 – May 2023) 29 months (N = 26,157)	Post-pandemic era (June 2023 – May 2024) 12 months (N = 11,667)
	N (%^a^)	N (%^a^)	N (%^a^)	N (%^a^)	N (%^a^)
**Age**					
50-54	27,594 (35.9)	6,212 (39.5)	8,188 (35.1)	9,498 (36.3)	3,696 (31.7)
55-59	19,797 (25.8)	4,205 (26.7)	6,093 (26.2)	6,567 (25.1)	2,931 (25.1)
60-64	15,798 (20.6)	3,154 (20.1)	4,848 (20.8)	5,342 (20.4)	2,454 (21.0)
65-69	9,751 (12.7)	1,564 (9.9)	3,003 (12.9)	3,423 (13.1)	1,760 (15.1)
70-75	3,923 (5.1)	599 (3.8)	1,172 (5.0)	1,326 (5.1)	826 (7.1)
**Sex** ^**b**^					
Female	34,624 (45.1)	7,127 (45.3)	10,491 (34.6)	11,800 (45.1)	5,206 (44.6)
Male	42,233 (55.0)	8,607 (54.7)	12,813 (63.1)	14,355 (54.9)	6,458 (55.4)
**Race** ^**c**^					
Asian	2,023 (2.6)	438 (2.8)	621 (2.7)	624 (2.4)	342 (2.9)
Black/African American	1,251 (1.6)	251 (1.6)	356 (1.5)	431 (1.7)	214 (1.8)
Hispanic/ Latino	62,041 (80.7)	12548 (79.8)	18780 (80.6)	21185 (81.0)	9528 (81.7)
Other	2,541 (3.3)	489 (3.1)	832 (3.6)	1033 (4.0)	187 (1.6)
Non-Hispanic/ Latino White	8,610 (11.2)	2008 (12.8)	2717 (11.7)	2801 (10.7)	1085 (9.3)
**Language Preference**					
English	26,425 (34.4)	5,701 (36.2)	8,072 (34.6)	8,901 (34.0)	3,751 (32.2)
Spanish	48,784 (63.5)	9,685 (61.6)	14,709 (63.1)	16,705 (63.9)	7,684 (65.9)
Other	1652 (2.2)	348 (2.2)	523 (2.2)	550 (2.1)	231 (2.0)
**Number of encounters (0–6+)**					
No visits	9.675 (12.6)	1,638 (10.4)	3,734 (16.0)	2,810 (10.7)	1,493 (12.8)
1–3 visits	30,505 (39.7)	9,121 (58.0)	7,687 (33.0)	8,317 (31.8)	5,381 (46.1)
More than 3 visits	36,682 (47.7)	4,975 (31.6)	11,884 (51.0)	15,030 (57.5)	4,793 (41.1)

^a^Percentages based on numbers of observations during each time interval.

^b^Excludes 11 patients with other/unknown sex (0 in pre-pandemic-, 1 in early-pandemic, 5 in vaccine-, and 5 in post-pandemic intervals).

^c^Excludes 395 patients with unknown race (0 in pre-pandemic-, 0 in pandemic, 83 in vaccine-, and 312 in post-pandemic intervals).

### Trend analysis

Monthly colorectal cancer screening rates declined from 9.3% pre-pandemic to 5.9% during the pandemic, with partial recovery to 7.5% in the vaccine era before declining again to 6.7% post-pandemic, though trend analyses showed no statistically significant differences across phases (p trend = 0.09; Table 2).

**Table 2:** Average colorectal cancer screening rates across pandemic-related time intervals weighted by number of months in interval, by screening modality.

	Pre-pandemic era (A)	Pandemic era (B)	Trend (A vs. B)	Vaccine era (C)	Trend (B vs. C)	Post-pandemic era (D)	Trend (C vs. D)
	N = 377,617 patient-months N (%)	N = 233,046 patient-months N (%)	P value*	N = 758,539 patient-months N (%)	P value*	N = 338,336 patient-months N (%)	P value*
Overall colorectal cancer screening	35,287 (9.3)	13,818 (5.9)	0.30	57,131 (7.5)	0.95	22,806 (6.7)	0.38
Screening modality							
Stool-based testing	30,876 (8.2)	12,634 (5.4)	0.40	51,626 (6.8)	0.90	19,842 (5.9)	0.41
FIT**	30,869 (8.2)	12,634 (5.4)	0.38	44,838 (5.9)	0.77	11,867 (3.5)	0.84
FIT-DNA test	7 (0.0)	0 (0.0)	0.65	6,788 (0.9)	0.07	7,975 (2.4)	**0.048**
Colonoscopy	4,377 (1.2)	1,169 (0.5)	**<0.0001**	5,470 (0.7)	0.06	2,945 (0.9)	0.08

*Based on interrupted time series regression; ** Fecal immunochemical test.

**Fig 1:**
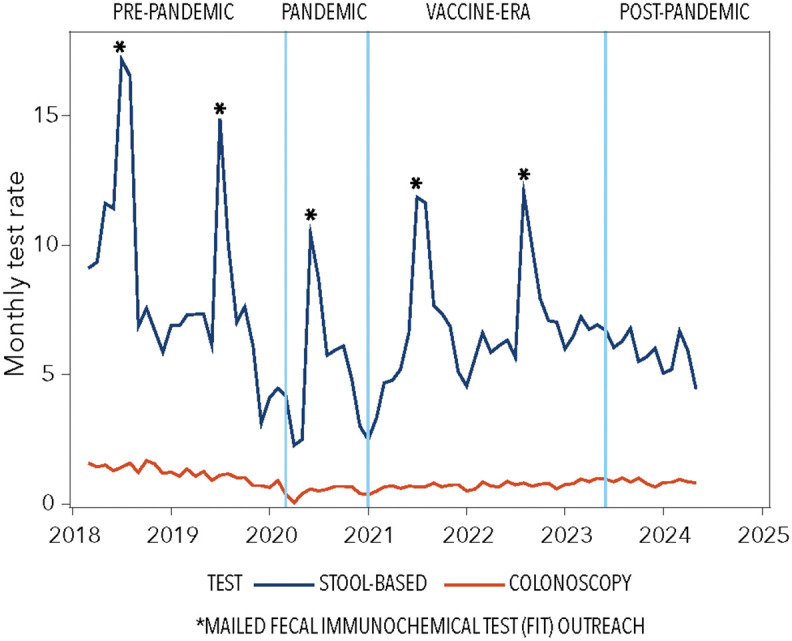
Trends in colorectal cancer screening during COVID-19-relevant time intervals, by screening modality (stool-based testing, colonoscopy). Legend: Plots depict monthly stool-based test (blue line) and colonoscopy (red line) rates across COVID-19-relevant phases: pre-pandemic (March 2018 – February 2020); pandemic (March – December 2020); vaccine (January 2021– May 2023); and post-pandemic (June 2023 – May 2024).

**Fig 2:**
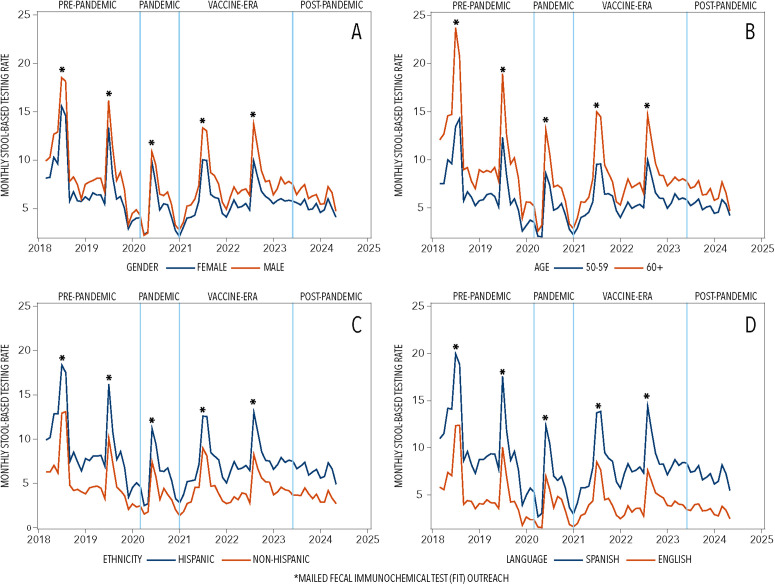
Trends in stool-based testing during COVID-19-relevant time intervals. Legend: Plots depict monthly stool-based testing rates across COVID-19-relevant time intervals: pre-pandemic (March 2018 – February 2020); pandemic (March – December 2020); vaccine (January 2021– May 2023); and post-pandemic (June 2023 – May 2024) by electronic health record derived characteristics: (A) sex (female, male), (B) age (50-59, 60+), (C) ethnicity (Hispanic, non-Hispanic), and (D) preferred language (Spanish, English).

**Fig 3:**
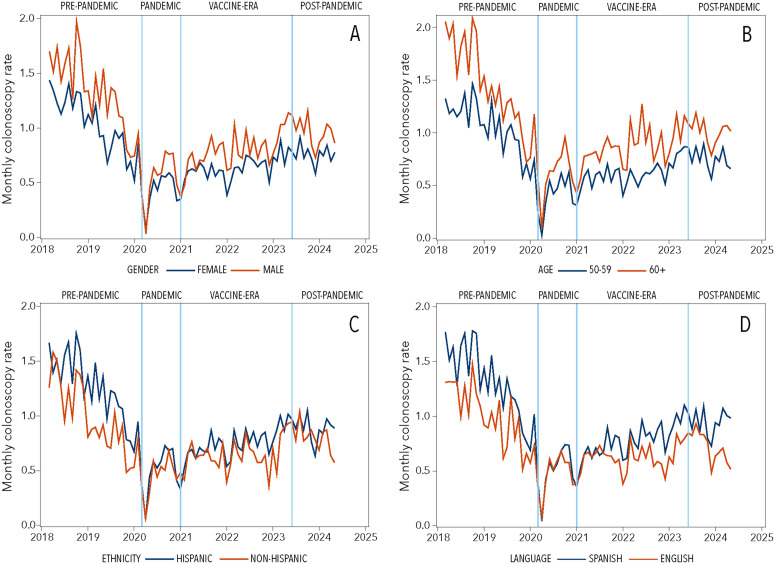
Trends in colonoscopy during COVID-19-relevant time intervals. Legend: Plots depict monthly colonoscopy screening rates across COVID-19-relevant timepoints: pre-pandemic (March 2018 – February 2020); pandemic (March – December 2020); vaccine (January 2021– May 2023); and post-pandemic (June 2023 – May 2024) by electronic health record derived characteristics: (A) sex (female, male), (B) age (50-59, 60+), (C) ethnicity (Hispanic, non-Hispanic), and (D) preferred language (Spanish, English).

Examining specific modalities, FIT usage followed a similar pattern with continued decline post-pandemic. FIT-DNA testing was introduced in July 2022 and showed significant growth from 0.9% to 2.4% (p = 0.048). Colonoscopy use dropped dramatically during the pandemic (1.2% to 0.5%, p < 0.001) and remained below pre-pandemic levels throughout subsequent periods. Average colorectal cancer screening across COVID-related time intervals by subgroups is provided in S1 Table. In both pre-COVID (March 2018 – Feb 2020) and post-COVID (July 2020 – May 2024) time intervals, average monthly screening rates were higher for males (vs. females), individuals aged 65–75 (vs. 50–64), Hispanic individuals (vs. non-Hispanic), and those preferring Spanish (vs. English). Monthly colorectal cancer screening rates by modality are illustrated in Fig 1; further stratification by sex, age, ethnicity, language preference is illustrated in Fig 2 for stool-based testing and Fig 3 for colonoscopy.

In post-hoc sensitivity analysis that assessed changes in monthly rates across pandemic-related phases, with the months of the mailed FIT outreach removed, we observed significant reductions comparing pre-pandemic and pandemic timepoints for overall colorectal cancer screening (8.1% vs. 4.9%; p for trend = .007) and FIT (6.9% vs. 4.4%; p for trend = .026). This suggests that mailed FIT outreach attenuated the impact of the pandemic on colorectal cancer screening rates. This analysis is shown in S2 Table. Global trends were not significant for any colorectal cancer screening (p trend = 0.09), stool-based testing (p = 0.12), or colonoscopy (p = 0.10).

### Geo-spatial analysis

**Fig 4:**
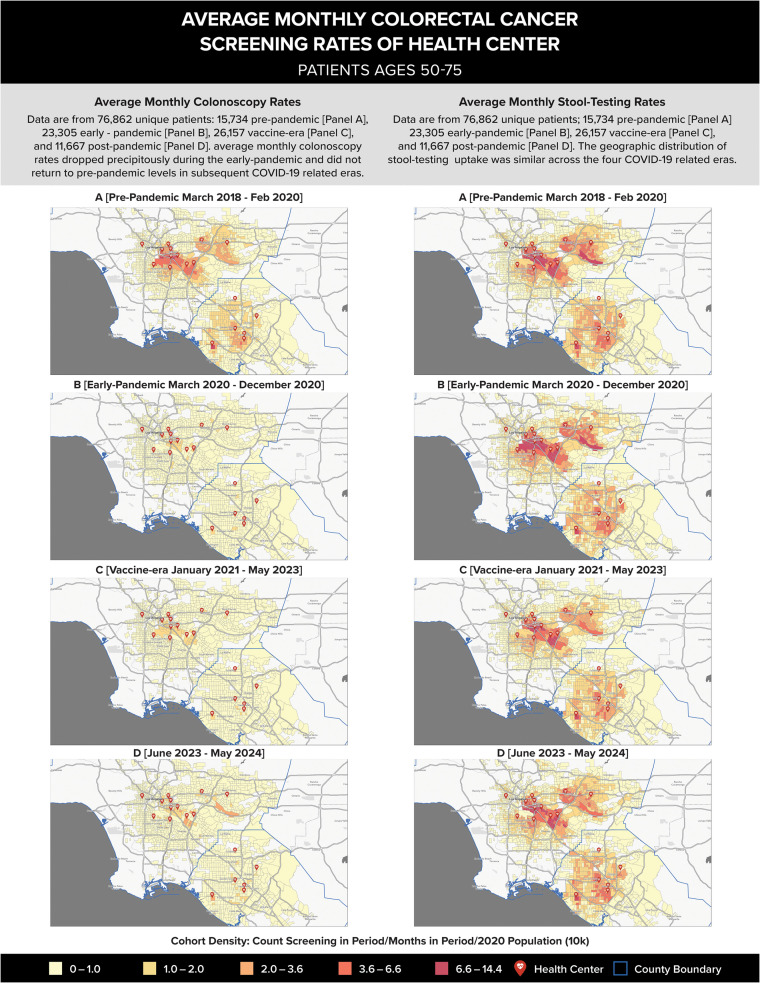
Average monthly colorectal cancer screening rates of health center patients ages 50−75. Legend: Left column panels A-D depict average monthly colonoscopy rates. Right column panels A-D depict average monthly stool-testing rates. Each panel shows average monthly colorectal cancer screening rates of health center patients across COVID-19-relevant phases: (A) pre-pandemic (March 2018 – February 2020); (B) pandemic (March – December 2020); (C) vaccine (January 2021– May 2023); and (D) post-pandemic (June 2023 – May 2024).

Fig 4 illustrates the spatial distribution patterns for patients who were eligible for colorectal cancer screening during the study period. The figure shows patient population density (number of colorectal cancer screening patients per population sized, based on the 2020 US Census) with the average monthly rates of colorectal cancer screening (stool-based testing versus colonoscopy) during our four COVID-19-relevant time intervals. Comparing average monthly stool-based testing proportions across the four time-periods (right four maps), no differences in trends were observed, suggesting minimal impact of COVID-19 on stool-based testing patterns. In contrast, spatial distribution patterns of colonoscopy (left four maps) showed marked reductions in the pandemic era, compared to pre-pandemic levels. Spatial distribution patterns show slight increases in colonoscopy use in the vaccine-availability and post-pandemic eras, but neither reach pre-pandemic levels.

## Discussion

We examined trends in overall colorectal cancer screening during four COVID-19-pandemic related phases (pre-pandemic, early-pandemic, vaccine era, and post-pandemic). Monthly screening rates in the vaccine era never returned to pre-pandemic levels and further declined even after the pandemic was declared over. Increases in stool-based testing aligned with mailed outreach campaigns, underscoring the value of home-based screening options during periods of disruption. Ongoing efforts are essential in FQHCs to promote colorectal cancer screening, maintain sufficient colonoscopy capacity, and close screening gaps to reduce the likelihood of delayed or missed colorectal cancer diagnoses.

While US national surveillance systems data for 2019–2022 show relatively rapid rebound in colorectal cancer screening rates [3,4,8,19,20], with most evaluations reporting rates meeting or exceeding pre-pandemic rates by 2022, our data shows a distinct pattern: Average monthly rates from April 2020 – December 2022 never reached pre-pandemic levels. This finding is consistent with Zhao and colleagues who reported a 2.8 percentage-point drop comparing 2019 (45.6%) with 2022 (42.8%), in analysis using US Uniform Data System data [9], and McGrath and colleagues who reported a 2.3 percentage-point drop in the same years (45.2% in 2019 and 42.9% in 2022), using EHR data from a subset of 200 FQHCs [13]. Two additional evaluations included FQHC data through 2021, both documenting post-pandemic screening gaps [14,15]. These findings confirm a widened FQHC screening gap compared to national levels (between 28–30 percentage points lower) for the years 2020–2022 [8–11,21–23].

We uniquely present data from 2022 to 2024, following the end of the public health emergency. Our analysis revealed rate declines in 2024 compared to 2022 levels, suggesting that sustained efforts may be necessary to address ongoing screening gaps. Our geospatial analysis identified locations where colonoscopy rates decreased following the pandemic, which may help pinpoint opportunities to assess and re-engage specialty providers capable of delivering colonoscopy services.

Notably, an additional operational challenge for health systems in the vaccine era was new US Preventive Services Task Force recommendations dropping the colorectal cancer screening initiation age to 45 years (from 50, the previous recommendation). Colorectal cancer screening rates for this expanded eligible population were reflected in the FQHCs’ quality reporting database in March 2022 (though not part of this study’s analyses) [24]. Given the younger patient populations they serve, FQHCs may need to invest greater effort to maintain screening levels under this expanded eligibility compared to health systems that primarily serve commercially insured or Medicare-enrolled patients. Future research might explore the new guideline’s impact on screening patterns in the 50–75 age range in FQHCs, adding to published reports based on national surveillance systems and commercially insured adults [25–27].

Despite the unique challenges faced by FQHCs, our subgroup analyses revealed that Hispanic and Spanish-preferring patients had higher colorectal cancer screening uptake than non-Hispanic and English-preferring patients. This finding may be attributed to our partnering FQHC serving a predominantly Hispanic/Latino population (≥80%) and providing culturally tailored resources, including translated materials and bilingual staff, which likely facilitated post-pandemic recovery in screening rates. Furthermore, since post-pandemic recovery was driven primarily by increases in stool-based testing rather than colonoscopy, the stronger preference for stool-based testing among Hispanics, as reported by Inadomi and colleagues, may help explain these findings [28].

Overall, our findings indicate that screening tests permitting self-collection at home, such as those for colorectal cancer, could mitigate the impact of future natural disasters on screening uptake and, consequently, on cancer diagnoses. As technology advances to detect early signs of cancer, attention should be paid to options that allow home-based self-collection. Nevertheless, home-based testing requires reliable infrastructure to lessen patient barriers to collection and return (e.g., reliable postal service and postage-paid options), systems to monitor program outcomes, and clinical resources to ensure that individuals receive their test results and that those with abnormal finding can obtain follow-up diagnostic procedures. Our team has previously documented low follow-up rates (33% − 44% within 1 year) in this and other health centers [29,30].

Apart from self-sampling tests, prior reports have recommended several strategies to lessen the impact of COVID-19 or related events. These strategies include prioritizing colonoscopy scheduling for higher-risk individuals, using risk-stratification approaches, increasing use of patient education approaches, and increasing use of automated reminders [31–33]. Others have recommended reducing structural barriers to care by bundling screening offers for multiple types of cancers, offering same-day screening (i.e., poop-on-demand), and expanding clinic hours [33,34]. Finally, telehealth and other digital solutions can be used to deliver health education and/or reminders to support screening uptake. These strategies warrant further investigation of how they can serve to maintain outreach during times of limited staff capacity and/or complement live sessions [32,33].

### Strengths and limitations

Our study has several strengths, including the large and diverse study population and our robust methods to evaluate stool-based-and colonoscopy screening rates. Our study also had limitations. Our analysis drew from one health center, whereas previous FQHC studies have examined broader regional or national networks [9,13–15]. Our partnering health center provides primary care services, and patients who have an abnormal fecal test result are referred to outside specialty care, and some colonoscopies are not recorded in primary care records. However, prior research with this health center shows high capture of follow-up colonoscopes in EHR data, compared to claims data for the same patients [29]. We were unable to remove from our denominator patients who had symptoms or were above average risk for colorectal cancer. Also, because we relied on clinic visits to define our population, we could not remove from our denominator people known to have left the health system or who were deceased. Finally, EHR data overwrites patient home address with the most current address; thus, maps may not reflect patient’s actual address for earlier intervals. Future research should compare our findings with analyses using other data sources (such as health plan claims data).

## Conclusions

Monthly post-pandemic screening rates never reached pre-pandemic levels and declined from 2022 to 2024. Sharp increases in stool-based testing coincided with mailed outreach events, highlighting the importance of home-based screening methods during disruptive events. In FQHC settings, sustained efforts are needed to promote colorectal cancer screening, ensure adequate colonoscopy access, and narrow screening gaps to reduce risks of delayed or missed diagnoses.

## Supporting information

S1 Fig The spatial distribution of cohort population density (health center patients) based on 2020 US census data among four COVID-19 time intervals. (TIF)

S1 Table Average monthly colorectal cancer screening rates among average-risk health center patients aged 50–75 (n = 83,256), by patient characteristics and for pre- and post-COVID time intervals. (DOCX)

S2 Table Average colorectal cancer screening rates across pandemic-related time intervals. (DOCX)

S1 File Copyright permission form signed for Fig 4. (PDF)
