# Supplementary material for: Temporal and geographic analyses of colorectal cancer screening during and after the COVID-19 pandemic in a federally qualified health center
Source: PLoS One. 2026 Mar 24;21(3):e0345248. doi: 10.1371/journal.pone.0345248 (PMC13012522; doi:10.1371/journal.pone.0345248)
Supplement: S1 Table — (DOCX) [file pone.0345248.s002.docx]

**S2 Table.** Average monthly colorectal cancer screening rates among average-risk health center patients aged 50-75 (n = 83,256), by patient characteristics and for pre- and post-COVID time intervals.

|  | Pre-COVID (March 2018 - Feb 2020) | | | Post-COVID (July 2020 - May 2024) | | | | |  |  |  |
| --- | --- | --- | --- | --- | --- | --- | --- | --- | --- | --- | --- |
| Characteristics | Number screened, Mean (SD) | CRC screening, Mean (SD), % | P-value comparing subgroup* | Number screened, Mean (SD) | CRC screening, Mean (SD), % | | P-value comparing subgroup* | | P-value comparing pre-COVID and post-COVID intervals* | Monthly percentage change (95% CI), %** |  |
| Overall | 1470 (542) | 9.6 (3.8) | NA | 1893 (560) | 7.1 (2.0) | | NA | | .0008 | 0.32 (0.08, 0.56) |  |
| Sex |  |  |  |  |  | |  | |  |  |  |
| Male | 888 (328) | 10.6 (4.2) | .0315 | 1154 (357) | 7.9 (2.3) | | <.0001 | | .0014 | 0.34 (0.07, 0.62) |  |
| Female | 582 (215) | 8.4 (3.4) |  | 739 (205) | 6.2 (1.6) | |  |  | .0006 | 0.29 (0.10, 0.49) |  |
| Age |  |  |  |  |  | |  | |  |  |  |
| 50-64 | 1135 (421) | 8.8 (3.5) | .0011 | 1368 (393) | 6.6 (1.8) | | <.0001 | | .0012 | 0.30 (0.09, 0.51) |  |
| 65-75 | 335 (126) | 13.7 (5.6) |  | 525 (172) | 9.2 (2.8) | |  |  | .0001 | 0.49 (0.15, 0.83) |  |
| Ethnicity |  |  |  |  |  | |  | |  |  |  |
| Hispanic | 1256 (453) | 10.5 (4.0) | .0003 | 1639 (477) | 7.8 (2.1) | | <.0001 | | .0005 | 0.34 (0.09, 0.59) |  |
| non-Hispanic | 214 (91.6) | 6.5 (3.0) |  | 234 (85) | 4.7 (1.6) | |  |  | .0043 | 0.24 (0.06, 0.43) |  |
| Language |  |  |  |  |  | |  | |  |  |  |
| English | 349 (148) | 6.3 (2.9) | <.0001 | 407 (133) | 4.6 (1.5) | | <.0001 | | .0045 | 0.22 (0.04, 0.39) |  |
| Spanish | 1095 (381) | 11.6 (4.3) |  | 1457 (427) | 8.5 (2.3) | |  | | .0004 | 0.38 (0.10, 0.65) |  |
| *based on Wilcoxon Rank Sum test | | | |  | |  | |  |  |  | |
| **based on Interrupted Time Series model, includes months from March 2018-Feb 2020 and July 2020-May 2024  CRC = colorectal cancer; SD = standard deviation | | | | | | | | | |  |  |
