# Supplementary material for: Temporal and geographic analyses of colorectal cancer screening during and after the COVID-19 pandemic in a federally qualified health center
Source: PLoS One. 2026 Mar 24;21(3):e0345248. doi: 10.1371/journal.pone.0345248 (PMC13012522; doi:10.1371/journal.pone.0345248)
Supplement: S2 Table — (DOCX) [file pone.0345248.s003.docx]

**S3 Table.** Average colorectal cancer screening rates across pandemic-related time intervals

|  | **Pre-pandemic era (A)** | **Pandemic era**  **(B)** | **Trend**  **(A vs. B)** | **Vaccine era**  **(C)** | **Trend**  **(B vs. C)** | **Post-pandemic era (D)** | **Trend**  **(C vs. D)** |
| --- | --- | --- | --- | --- | --- | --- | --- |
|  | N = 314,077 patient-months  N (%) | N = 185,842 patient-months  N (%) | P value* | N = 650,863 patient-months  N (%) | P value* | N = 338,336 patient-months  N (%) | P value* |
| Overall colorectal cancer screening | 25,304 (8.1) | 9,045 (4.9) | **<0.01** | 44,125 (6.8) | 0.73 | 22,806 (6.7) | **0.03** |
| Stool-based  testing | 21,726 (6.9) | 8,120 (4.4) | **0.02** | 39,395 (6.1) | 0.83 | 19,842 (5.9) | **0.04** |
| FIT** | 21,720 (6.9) | 8,120 (4.4) | **0.03** | 33,055 (5.1) | 0.27 | 11,867 (3.5) | 0.59 |

Rates were weighted by number of months in interval, by screening modality, and excluding months of mailed fecal test outreach; *Based on interrupted time series regression; ** Fecal immunochemical test
