# Supplementary material for: Temporal and geographic analyses of colorectal cancer screening during and after the COVID-19 pandemic in a federally qualified health center
Source: PLoS One. 2026 Mar 24;21(3):e0345248. doi: 10.1371/journal.pone.0345248 (PMC13012522; doi:10.1371/journal.pone.0345248)
Supplement: S1 File — (PDF) [file pone.0345248.s004.pdf]

Request for Permission to Publish Content under CC-BY License

Dear Rights Holder or Representative,

I have submitted a paper for publication in a PLOS journal, and wish to include the content listed below in the paper. I'm hereby requesting your (or your company's or institution's) permission to include the content in my paper. Please note that all PLOS journals are published under a Creative Commons Attribution License (CC BY), which allows for unrestricted use and distribution, even commercial, as long as attribution is given to the creator or rights holder of the content. See https://creativecommons.org/licenses/by/4.0/.

To grant me permission to use the content in my PLOS paper, please fill in the information below and then scan the completed form and send it to me at my email address.

Thank you.

My name:

Gloria D. Coronado, PhD

My email address:

gdcoronado@arizona.edu

Description of the content which I'm seeking permission to use (citation and/or title, and pasted screen shot, if applicable):

maps of population density and of colorectal cancer screening in Los Angeles and Orange county California. Maps are displayed in Figure 4 of paper, Temporal and Geographic Analyses of Colorectal Cancer Screening During and After the COVID-19 Pandemic in a Federally Qualified Health Center

Link to the Content:

* * *

On behalf of myself or the rights holder, I hereby grant the permission sought herein.

Signature of Party Granting Permission:

Date:

2026-03-10

Printed Name and Title:

Jackson L. Voelkel, MUS, Data Reporting and Analytics Consultant V
